# Effect of dye complex structure on performance in DSSCs; An experimental and theoretical study

**DOI:** 10.1016/j.heliyon.2022.e11692

**Published:** 2022-11-17

**Authors:** Faezeh Arjmand, Zohreh Rashidi Ranjbar, Hassan Fatemi E. G

**Affiliations:** a Department of Chemistry, Faculty of Sciences, Shahid Bahonar University of Kerman, Kerman, Iran; b Faculty of Physics, Shahid Bahonar University of Kerman, Kerman, Iran

**Keywords:** Dye sensitized solar cells, Bandgap, Cobalt (ІІ) complexes, Theoretical studies

## Abstract

Use of cobalt-complexes as dye in dye sensitized solar cells (DSSCs) has been a viable contender in the field of photovoltaics due to their low cost, easy production, and wide availability of sources. In this study, we investigated the effect of succinic acid (suc), 1,10-phenanthroline (phen), and benzene-1,3,5-tricarboxylate (BTC) as ligand in metals complex sensitizers with these general formulas: (1) [Co(suc)]_n_, (2) [Co_2_(suc)_2_(phen)_2_], (3) [Co_3_(BTC)_2_(H_2_O)_n_]_n_, and (4) [Co(HBTC) (phen) (H_2_O)_2_]; for DSSCs. The bandgap, and energy levels have an important role in photoelectron emission during photovoltaic processes. The bandgap, and energy levels of these dyes (1–4) were estimated by Ultraviolet-Visible (UV-Vis), spectroscopies, and cyclic voltammetry (CV).

Delocalized π-electron of phenanthroline in two compounds (2, 4) by effectively reducing band gap energies leads to power conversion efficiency (PCE) of 0.511%, and 1.006%, respectively. Their PCEs to compounds (1, 3) showed an approximate increase of 60%, and 31%, respectively. Furthermore, BTC ligands by more coordinated carboxylate groups in contrast with succinic molecules due to binding sites with TiO_2_ cause further enhancement in the efficiency for compounds (3, 4) in contrast with compounds (1, 2). Reasonable agreement is found between experimental and theoretical values calculated using density functional theory (DFT) for the bandgaps and energy levels.

## 1 Introduction

The study of solar energy is one of the critical fields in energy development [1, 2]. Unfortunately, fossil fuels still continue to have a high percentage of use as a primary energy source [3]. Nowadays, the world has considered the fact that these fuels increase greenhouse gas emissions, and cause global warming [4]. Therefore, alternative energy sources with pollution-free, and limitless options have received significant emphasis worldwide. Solar energy is an important primary, renewable and alternative source that converts to electricity via the photovoltaic effect [5, 6, 7].

Dye-sensitized solar cells (DSSCs) are among the third generation of solar cells, offering a promising alternative to conventional solar cells due to their low cost, easy fabrication and abundant availability [8, 9, 10, 11, 12]. Among the first applications of DSSCs was Grätzel and O'Regan in 1991. The studies showed both organic and inorganic dyes use in DSSCs due to their delocalized π-electrons having excellent conductivity. However, inorganic dyes have more durability and thermal stability compared to organic dyes. Use of some dyes such as organometallic compounds and transition metal complexes therefore have both advantages [10, 11, 12].

Transition metal complexes have broad absorption spectra from Near-Infrared (Near-IR) to UV regions and excellent light-harvesting capability. Inorganic complexes could therefore carry out the sensitizer role in DSSCs. Since these structures have multi-dentate organic ligands, a better bond occurs between the dye molecules and photo-anode surface. Some transition metal complexes have been used as inorganic dyes or electrolytes, and others in counter electrode fabrication. Use of transition metal complexes as solid electrolytes or dye in DSSCs structures can improve stability and photovoltaic performance [13, 14, 15, 16, 17].

In recent years, among investigated dyes, ruthenium complexes consisting of thiocyanate ligands have demonstrated acceptable performance [18]. However, ruthenium is an expensive and rare metal which is a drawback in the design of low-cost DSSCs. The favorable photovoltaic, and broad absorption spectra properties and low-cost of earth abundant-metal complexes (based on Zn, Cd, Ni, Fe, Cu, Co, etc.) have made them great candidates for DSSCs [19, 20, 21, 22]. Economic, photo-physical, and environmental considerations make cobalt complexes attractive alternatives to ruthenium. However, transition metal complexes have unique structural features such as processability, obtainability of several organic molecules, low cost, and easy synthesis, etc. 1,10-phenanthroline (phen) is redox indicator and the strong chelating agent for many metal ions by its two nitrogen donors, that has been known for many years. Phen complexes have wide application areas due to their high light emissions at both UV and Vis regions, high charge transfer mobility, and good electro/photo active properties.

More recently, novel theoretical studies have been completed to estimate the band gap, open-circuit voltage (V_OC_) values in organic solar cells. Also, photovoltaic and optoelectronic properties such as absorption spectrum (with solvent or gas phase condition), electrons density, electron transport contributions (% ETC), excitation energies, and oscillating strength, have been computed using level of density functional theory (DFT). These computational data help the experimental designing of solar cells fabrication [23, 24, 25].

In this study, we synthesized and characterized four cobalt (II) complexes (1–4), and used them as inorganic-organic dyes to fabricate DSSCs. Short-circuit current density (J_SC_), open-circuit voltage (V_OC_), and fill factor (FF) were calculated for (1–4) DSSCs, experimentally. The results show dye [Co(HBTC) (phen) (H_2_O)_2_](4) has a higher power conversion efficiency that is related to having two factors; electron-donating ligand (phen), and one uncoordinated group (HBTC). Also, UV-Vis spectroscopies, and CV analysis were used to calculate the bandgap energies, Lowest Unoccupied Molecular Orbital (LUMO), and Highest Occupied Molecular Orbital (HOMO) levels. Furthermore, DFT calculations were conducted to reveal bandgap energies, and HOMO/LUMO levels.

## 2 Experimental

### 2.1 Material and methods

H_2_suc (succinic acid), phen, H_3_BTC, CoCl_2_.6H_2_O, ethylene glycol, I_2_, potassium iodide, potassium chloride, acetic acid, ethanol, N,N-dimethylformamide **(**DMF), dimethylsulfoxide (DMSO), acetonitrile, and ethanol were purchased commercially from Sigma-Aldrich Co. Fluorine-doped tin oxide (FTO) glass Plates (15–20 Ω/square) were obtained from Sharif University of Technology. TiO_2_ nanoparticles (P25, 20 nm) were purchased from the Iranian Nano Materials Co. FT–IR spectra were recorded as KBr pellets with a Brucker Tensor 27 spectrophotometer, and a Philips Co. diffractometer was used to analyze the phase purity of the obtained product. The electrochemical cyclic voltammetry (CV), and impedance measurement (EIS) of complexes were obtained by an AutoLab instrument (302N Potentiostat, Netherlands). The photocurrent density-voltage (J-V) performance of the PSCs was taken by a Solmetric I–V Curve Tracer (NanoSAT Co., Iran) and an Auto adjustable solar simulator (Karmana Photonics, Iran with an AM of 1.5, 100 mW/cm^2^). The absorption spectrophotometer and DFT calculations were investigated by Optizen 3220 and Gaussian 09W software, respectively. Incident Photon to Current Efficiency (IPCE) analysis was performed using a silicone NP photodiode (IPCE-020, Sharif Solar Co., Iran).

### 2.2 Synthesis of dyes

#### 2.2.1 Synthesis of (1) [Co(suc)]_n_

5 mL pink aqueous solution of CoCl_2_.6H_2_O (0.24 ​g, 1 ​mmol) was prepared. This solution was slowly added to the aqueous solution (3.5 ​mL) of succinic acid (0.03 g, 0.25 ​mmol) under reflux condition for 2 ​h [26]. The resulting solution was filtered and used for a photo anode of dye solar cell immersion. Melting point ​= ​376°°C, IR (KBr, cm^−1^) selected bands: 3406 (ν_O–H_); 3134 (ν_C–H_); 1619 (ν_as(COO_^-^_)_); 1405 (ν_s(COO_^-^_)_); 1427, 1304 (ν_C–H_); 1193, 1078, 997 (ν_C–C,C_–_O_); 915, 805, 641 (ν_CH2_); 462 (v_Co–O_), CHN (%):calculated. for C_4_H_4_CoO_4_(175): C 27.43, H 2.29; found: C 26.81, H 1.87.

#### 2.2.2 Synthesis of (2) [Co_2_(suc)_2_(phen)_2_]_n_

CoCl_2_·6H_2_O (0.24 ​g, 1 mmol) was dissolved in 10 mL distilled water, and this solution was added to 10 mL ethanolic solution succinic acid ligand (1 mmol, 0.12 g). Then the mixture was added to a 10 mL ethanolic solution of phen (0.2 g, 1 mmol). A gray powder precipitated and then dissolved in DMF [27]. The resulting pink solution was filtered, and a photo anode of dye solar cell was immersed in this solution. Melting point = 315 °C, IR (KBr, cm^−1^) selected bands: 3444 (ν_O–H_); 2924, 2851 (ν_C–H_); 1672 (ν_as(COO_^-^_)_); 1380 (ν_s(COO_^-^_)_); 1578 (ν_C=N_); 1420, 1340 (ν_C–H_); 1202, 1082, 960 (ν_C–C_, _C___O_); 827, 780 (ν_CH2_); 636,420 (ν_Co–O_, _N_), CHN (%):calculated for C_32_H_24_Co_2_N_4_O_8_ (710): C 54.08, H 3.38, N 7.89; found: C 53.84, H 3.21, N 7.68.

#### 2.2.3 Synthesis of (3) [Co_3_(BTC)_2_.12 H_2_O]_n_

The Co(NO_3_)_2_.6H_2_O (0.29 g, 1 mmol) was dissolved in DMF (7.5 mL), and poured dropwise into a prepared 10 mL ethanolic solution of BTC (0.105 g, 0.5 mmol), and some powders were isolated. By adding 5 mL DMF to participation powder, the colloidal solution was obtained [28], and a photo anode of dye solar cell was immersed in this solution. Melting point = 257 °C, IR (KBr, cm^−1^) selected bands: 3256 (ν_O–H_); 2929 (ν_C–H_); 1717 (ν_as (COO_-_)_); 1382 (ν_s (COO_^-^_)_); 1431, 1323 (ν_C–H_); 1221, 1104, 993 (ν_C–C, C___O_); 905, 739, 599 (ν_CH2_); 418 (ν_Co–O_), CHN(%):calculated for C_18_H_30_Co_3_O_24_ (807): C 26.77, H 3.72; found: C 26.53, H 3.51.

#### 2.2.4 Synthesis of (4) [Co (HBTC) (Phen) (H_2_O)]_n_

An aqueous solution of CoCl_2_.6H_2_O (1 mmol, 0.24g) was prepared, and then it was added dropwise to a 10 mL alcoholic solution of BTC (0.195 g, 1mmol). Then, the mixture was added to a 10 mL alcoholic solution of phen (0.2 g, 1mmol) under reflux condition for 24 h, and an orange powder was obtained [29]. The precipitation was dissolved with 5 mL DMF and used to immerse a photo anode of dye solar cell. Melting point = 257 °C, IR (cm^−1^, KBr) selected bands: 3428 (ν_O–H_); 3061 (ν_C–H_); 1703 (ν_as(COO_^-^_)_); 1344 (νs_(COO_^-^_)_); 1599 (ν_C=N_): 1425 (ν_C–H_); 1224, 1103, 930 (ν_C–C, C___O_); 848, 779 (ν_CH2_); 523,422 (ν_Co–O, N_). CHN (%): calculated for C_21_H_14_CoN_2_O_7_ (465): C 54.19, H 3.01, N 6.02; found: C 53.78, H 2.73, N 5.86.

### 2.3 Fabrication of DSSC

**Scheme 1:**
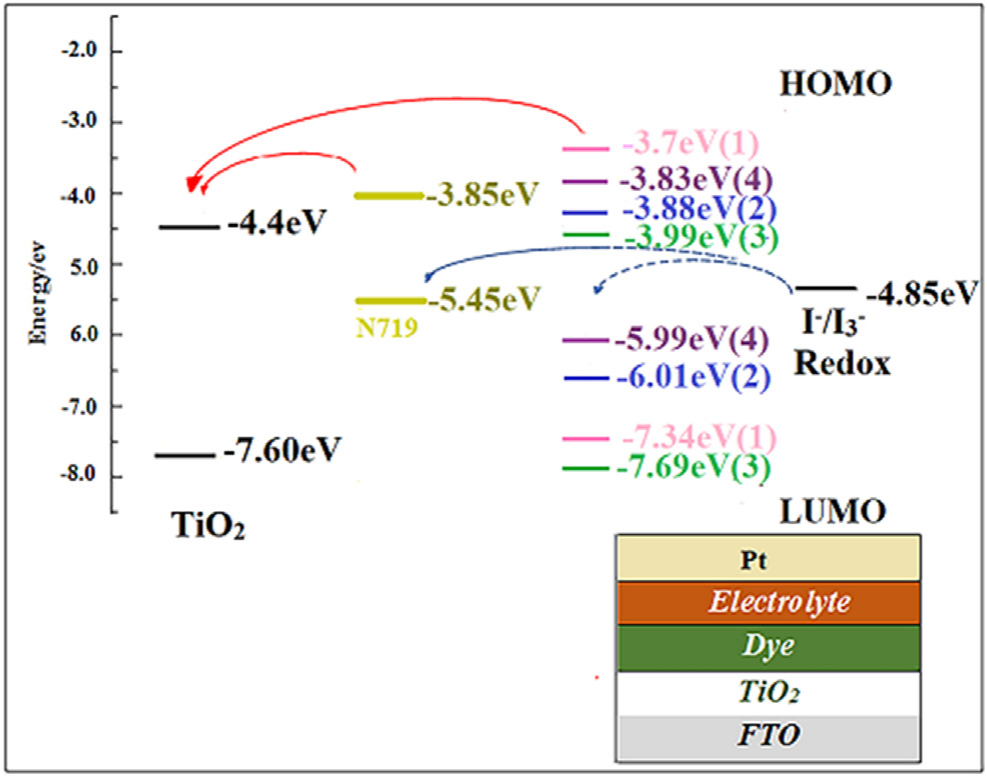
The structure of devices, and conduction band edge alignment of TiO_2_ and dyes (1–4) and N719.

The structure of the solar cells is shown in Scheme 1. All DSSCs (1cm^2^ active area) were assembled according to the following procedure: FTO glasses (1.0 cm ∗1.0 cm) were used as substrates and cleaned sequentially with detergent, deionized water, acetone, and ethanol under sonication for 10 min. To prepare a photo-anode, 1.0g TiO_2_ was mixed in 1.5 mL acetic acid, and coated onto FTO glass film by the Doctor Blade technique. The photo-anode was then calcined at 500 °C for 30 min. The prepared electrodes were immersed in cobalt (II) complexes (1–4) solution (0.01 mM in ethanol) for 24 h at room temperature. The counter electrode of Pt was used and assembled with a photo-anode. All DSSCs were filled with I^−^/I_3_^-^ redox couple electrolyte, consisting of I_2_ (0.183 g) and potassium iodide (2.07 g) in ethylene glycol (25 mL).

### 2.6 Theoretical studies

DFT method with the B3LYP function was applied for the geometry optimization, and HOMO/LUMO energy levels calculations. The initial geometries for calculation were taken from X-ray crystal structures (1–4). The crystal structures were obtained from the Cambridge Crystallographic Data Centre (http://www.ccdc.cam.ac.uk/conts/retrieving.html). The complexes were previously prepared, and their structures was characterized (CCDC: 830032, 602265, 921721 and 171923 for complexes 1–4, respectively) [26, 27, 28, 29].

## 3 Results and discussion

### 3.1 Optical absorption spectroscopies

The bandgaps of dyes (1–4) were estimated by absorption spectra. The absorption data is obtained from UV-Vis spectroscopies. We prepared a DMSO solution (0.001 mM, 20 mL) of complexes for absorption analysis spectra were acquired at room temperature. Also, absorption bands are extended from 200 nm to 800 nm wavelengths and corresponding wavelengths to the bandgaps were calculated by optical absorption data, and Tauc plots [30].

#### 3.1.1 The photo-absorption of dyes (1–4)

**Figure 1(a–d):**
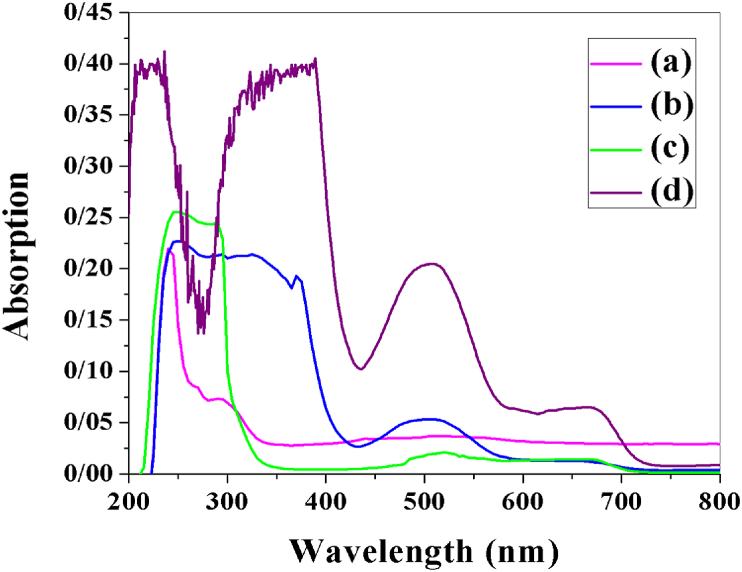
Absorption spectra of dyes 1–4.

Figure 1(a-d) shows absorption spectra of complexes 1–4. In UV–Vis absorption spectrum of complex 1 absorption peak at 235 nm is assigned to π→π∗ ligand internal electron transition. The peak at 270 nm is attributed to ligand-to-metal charge transfer (LMCT) electron transition. Also, complex 1 has a tetrahedral structure (Td), and two peaks at 500, and 640 nm are assigned to d→d electron transitions of Co (II) ions; ^4^A_2_→^4^T_1_(P) ^4^A_2_→^4^T_1_(F) (Figure 1a) [26, 30].

UV–Vis absorption spectrum of 2 has a peak at 250 nm and is assigned to electron transition oπ→π∗, and peaks at 325–370 nm are assigned to LMCT (O→Co and, N→Co) (Figure 1 b). The two different absorption bands at 255 nm and 290 nm in the UV–Vis absorption spectrum of 3 are assigned to π→π∗ transition, and LMCT (O→Co), respectively (Figure 1c). Also, two peaks at 245 nm and 330 nm are assigned to the transition of π→π∗, and LMCT (Figure 1 d) in complex 4. Since the Co (II) (d^7^)complexes (2–4) have octahedral structures, the peaks at 500–655 nm are assigned to d→d electron transitions from the excited-state ^4^T_1_g (F) to the ^4^A_2_g, and ^4^T_1_g(P) (Figure 1b-d) [26]. Since the results of UV–Vis absorption affect the efficiency of solar cells; solar cells prepared by phen complexes will have higher efficiency.

### 3.2 Optical band gap evaluation from UV-Vis

The band gap energy (Eg) are evaluated based on Tauc method and the theory of optical absorption [31]. The band gap energies of dye complexes 1–4 are calculated by Eq. (1) for a direct band gap of charge carried from allowed energy levels.

(1) $${\left(\alpha h\nu \right)}^{\frac{1}{r}}=A\left[h\nu -E_{g}\right]$$

where *E*_*g*_*, ν, h, A*, and *α* are the bandgap of the semiconductor, the frequency of incident photons, the plank's constant, a constant and absorption coefﬁcient, respectively. The value of an index representing r in the above relation expresses the nature of optical absorption; for example, 1/2, 2, 3, and 3/2 are presented as the direct allowed, indirect allowed, indirect forbidden, and directly forbidden transitions, respectively. In this regard, the optical bandgap was calculated for direct allowed transition (r = 1/2), and the plots variation between (αhv) [2] and (hv) in eV. The bandgap values of dyes 1–4 can be extracted from extrapolation of Tauc plots and are calculated as 3.64 eV, 2.13 eV, 3.7 eV, and 2.16 eV, respectively (Fig. S1 a-d). All results are listed in Table 1.

**Table 1:** Characteristic data for dyes (1–4) and N719: LUMO and HOMO levels, E_red_ (reduction peak from the CV), Eg (the calculated band gap from the Tauc diagram).

Dye	E_(red)_ (V)	HOMO (eV)	LUMO (eV)	Eg(eV)
1	-1	-7.34	-3.7	3.64
2	-0.84	-6.01	-3.88	2.13
3	-0.73	-7.69	-3.99	3.7
4	-0.89	-5.99	-3.83	2.16
N719	----------	-5.45	-3.85	1.6

### 3.3 Spectroscopic characterization of dye complexes (1–4)

**Figure 2(a–d):**
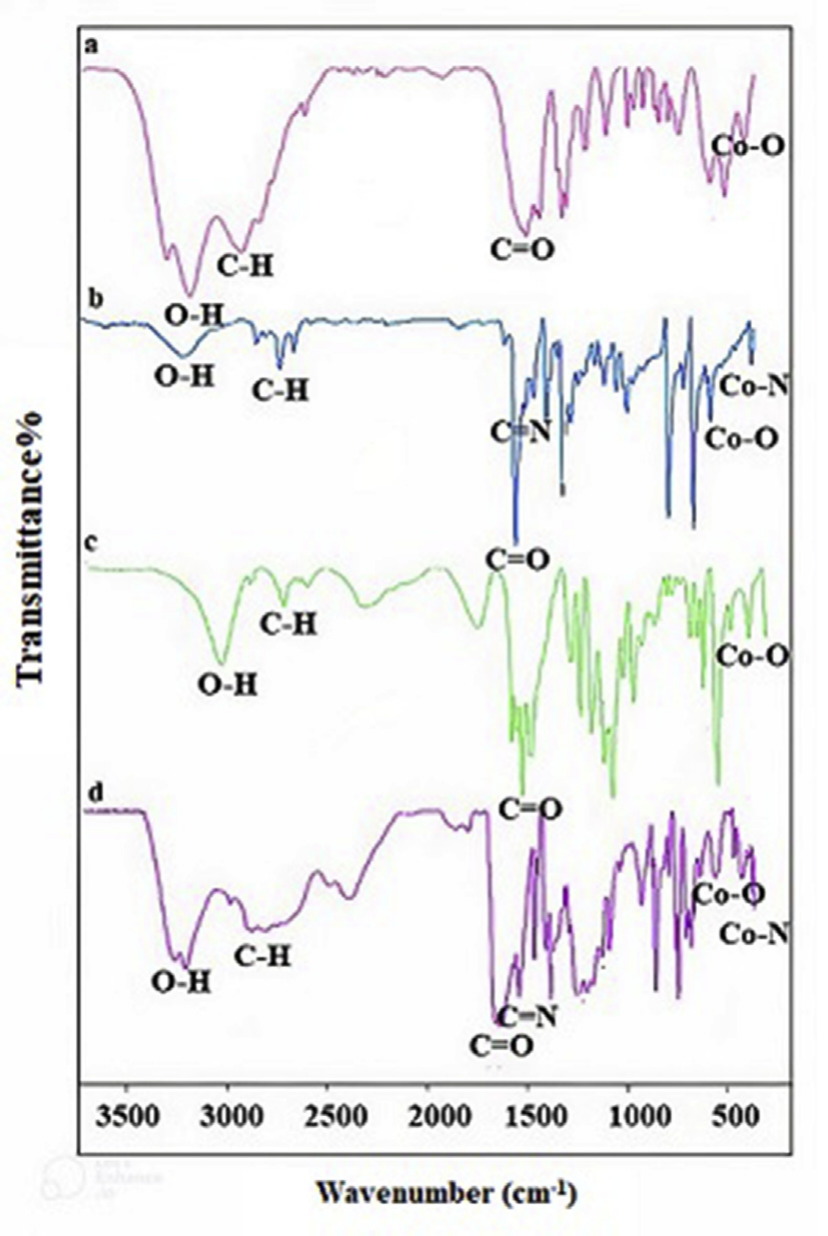
The IR spectra of dyes (1–4) from cobalt (II) complexes.

The infrared spectra of dyes (1–4) show some harmonic vibrational frequencies in the range of 400–4000 cm^−1^ (Figure 2 a-d). A broad peak of O–H appears in 3200–3500 cm^−1^, and relatively medium absorption bands at 2800-3100 cm^−1^ are assigned to C–H modes. Strong bands in the region of 1600–1700 cm^−1^ and 1300–1400 cm^−1^ correspond to asymmetric and symmetric vibrational modes of C=O bonds. Other bands at 1570-1600 cm^−1^ are due to vibrational modes of C=N bonds, and bands at 900-1200 cm^−1^ are related to the vibration of C-X (X: C, O) bonds. Also, some observed bands at 400-600 cm^−1^ correspond to the Co-X (X: O and N) interactions in the structures [32].

### 3.4 X-ray diffraction (XRD) analysis of dyes (1–4)

**Figure 3:**
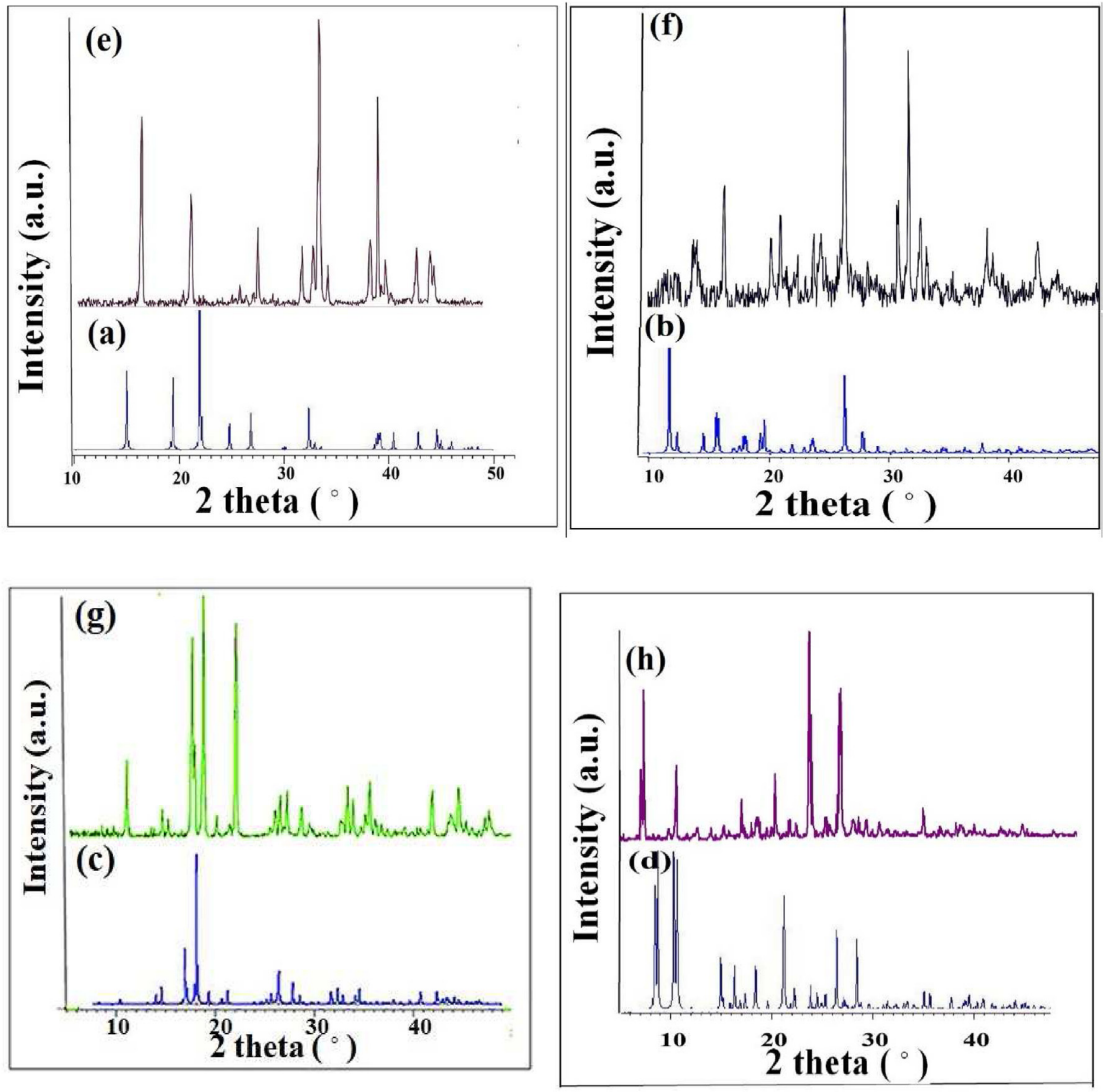
XRD patterns of complexes (1–4): a-d) simulation and (e–h) experimental.

XRD analysis for phase identification and crystallinity was performed. The XRD patterns of complexes (1–4) are shown in Figure 3(a-d), respectively. In this case, the reflections are almost sharp due to the excellent crystallinity. Figure 3(e-h) indicates the XRD patterns of complexes (1–4) where peaks resemble the simulated patterns from single X-ray crystallography data [26, 27, 28, 29]. No reflection peaks of impurities were observed, proving the products have high purity.

### 3.5 Electrochemical measurements

Electrochemical redox potentials for 1–4 complexes (0.01 M) were obtained by CV using a three-electrode cell, with Pt electrodes as the counter, carbon electrode as the working electrode, and an Ag/AgCl as the reference electrode in KCl (0.1 M) as a supporting electrolyte with 0.1 V/s scan rate [33]. To determine the possibilities of electron injection from the valence band (VB) to the conduction band (CB) of dyes, and electron drift in four cells, HOMO and LUMO energy levels were measured by cyclic voltammetry data.

#### 3.5.1 Cyclic voltammetry analysis and HOMO/LUMO levels calculation

**Figure 4:**
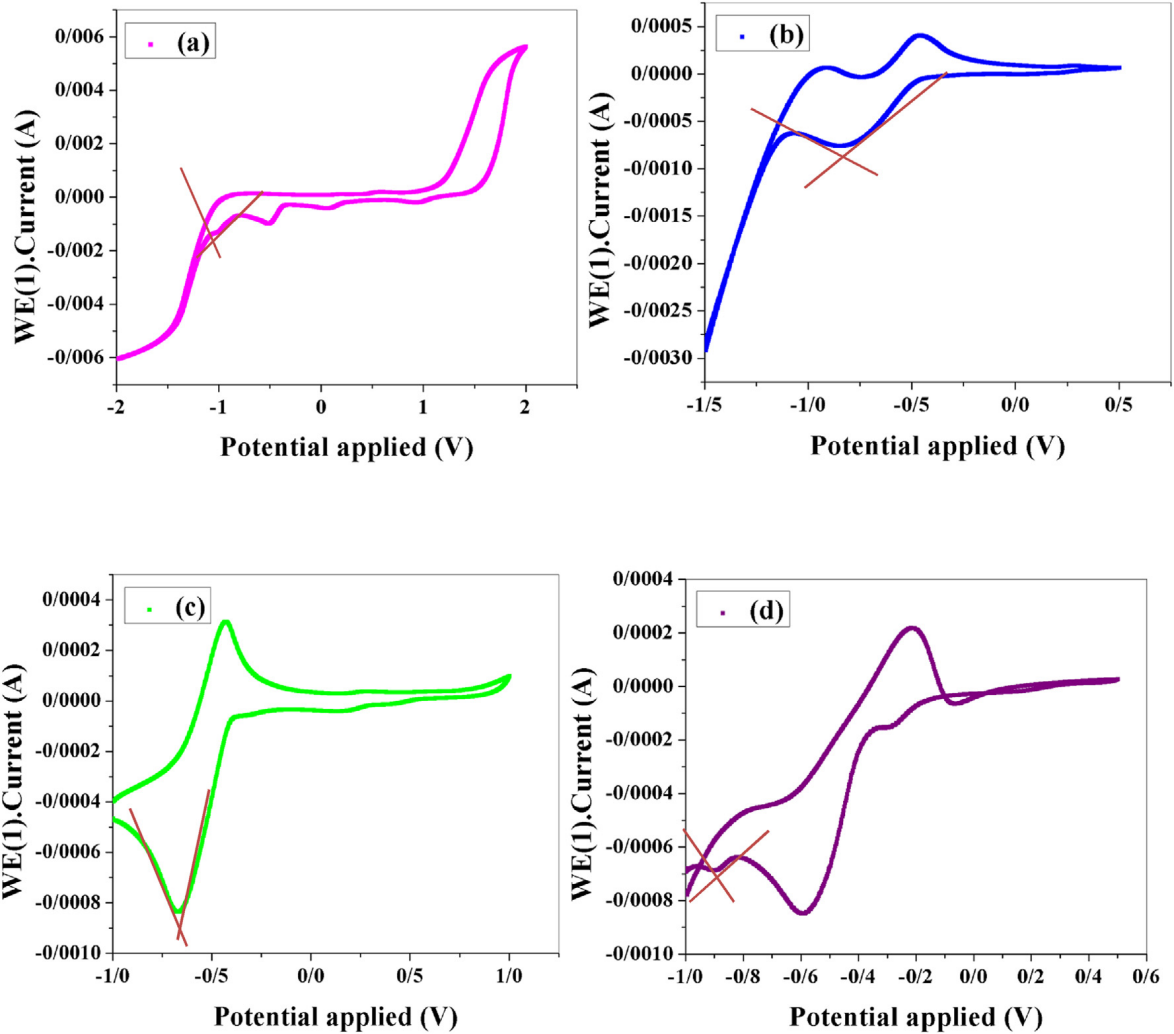
(a–d) Cyclic voltammetry graphs of dyes 1–4 (10 mM solution in dry acetonitrile) measured at a scan rate of 100 mV/s.

From the CV curves of 1–4, reversible behaviors are observed (Fig. 4a-d). Assuming that the energy levels of Ag/AgCl in saturated KCl is 4.7 eV; the LUMO levels were estimated from the CV data, E_LUMO_ ​= ​−(E_red_ ​+ ​4.725) eV, E_red_ is onset reduction potential waves, and E_HOMO_ ​= ​E_LUMO_-Eg, Eg is bandgap energy [34, 35]. Table 1 summarizes the results analyzed from the CV of 1–4. The HOMO, and LUMO energy levels values of the dyes are significant parameters in DSSCs. It is desired that the LUMO energy levels value must be above the conductivity band of the TiO_2_ semiconductor, and the HOMO energy levels value of the dye must be below the redox potential of the redox electrolyte to spontaneously working in the solar cells. The energy diagrams of dyes (1–4), and conduction band edge alignment of TiO_2_ nanoparticles are illustrated in Scheme 1. The all-synthesized complexes have suitable properties for dye-sensitized solar cells.

### 3.6 DFT calculations and HOMO/LUMO measurements

**Figure 5:**
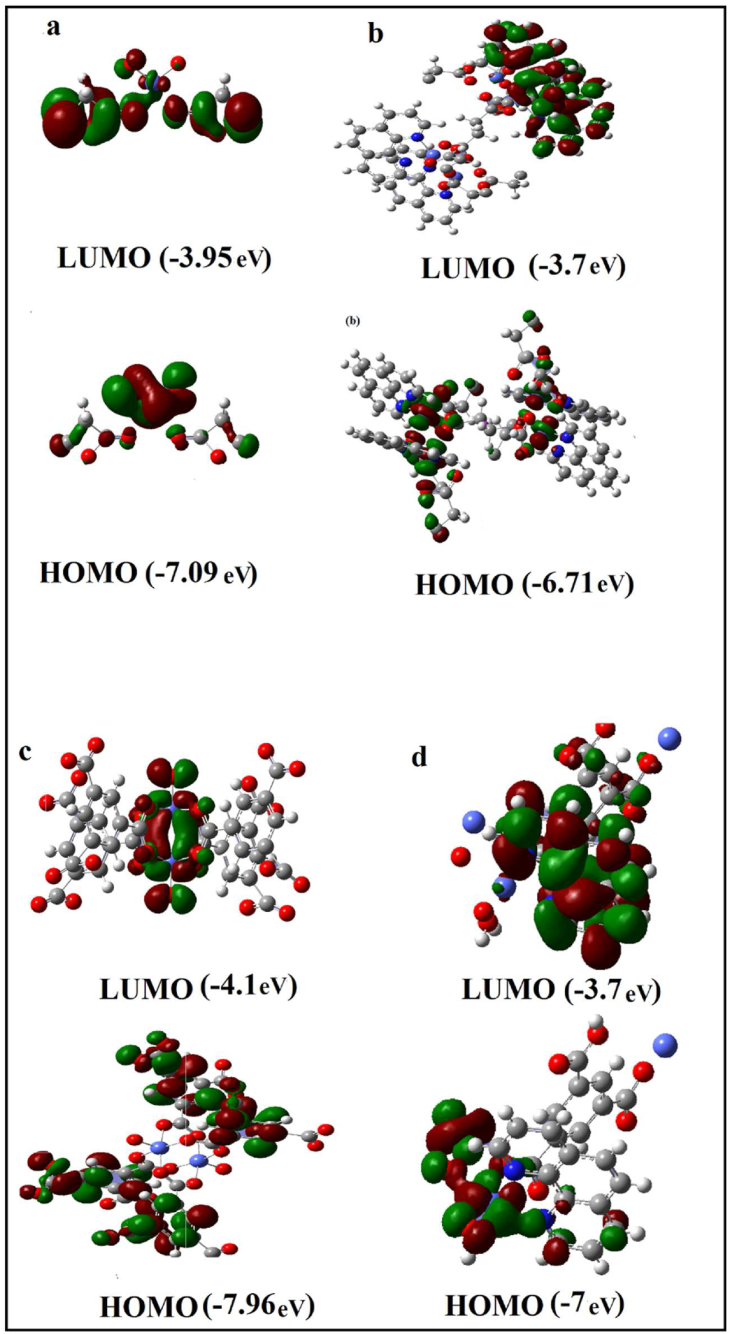
The bandgap, energy levels, and the frontier molecular orbitals of the HOMO and LUMO (a–d) of dyes (1–4) calculated with DFT at B3LYP/LanL2DZ level of theory.

The calculated frontier molecular orbitals (HOMO, and LUMO), and electron densities distribution of complexes 1–4 are shown in Figure 5, where the computational calculations are performed using Gaussian 09 software. The redistribution of electron densities shows the pronounced push-pull characteristics of the dye, giving an efficient intramolecular charge separation. DFT calculations lead to bandgap energies at 3.14 eV, 3.01 eV, 3.86 eV, and 3.30 eV, which correspond to excitations from HOMO to LUMO. The LUMO is mostly found in electron withdrawing groups through the π-spacer, while the HOMO is primarily found on the electron donating group and π-spacer. The four cobalt complexes have undergone theoretical computations to disclose the theoretical prediction of their effectiveness as sensitizers in DSSC. The outcomes of the calculations show that the COOH functional group is a significant element that has an impact on the DSSC's electron transport activity. As a result, during the transfer of electrons, the complexes might act as the medium through the COOH. On the other hand, the DSSC may benefit more from the complexes 2 and 4 with the phen ligand than from molecules 1 and 3. These findings ought to be useful in the development of novel sensitizers for DSSCs.

As compared to experimental gap energy values in the solution, the theoretical transition energies have a little different because of the calculation have mathematical constructions in the gas phase rather than description of single-particle states. Since, band gap is dependence on particle size, computed band gap of one molecule in the gas phase is not perfect value in comparison with experimental. Therefore, some DFT-computed calculation such as band gaps have limited, due to systematically incorrect estimate of values [36, 37].

**Figure 6:**
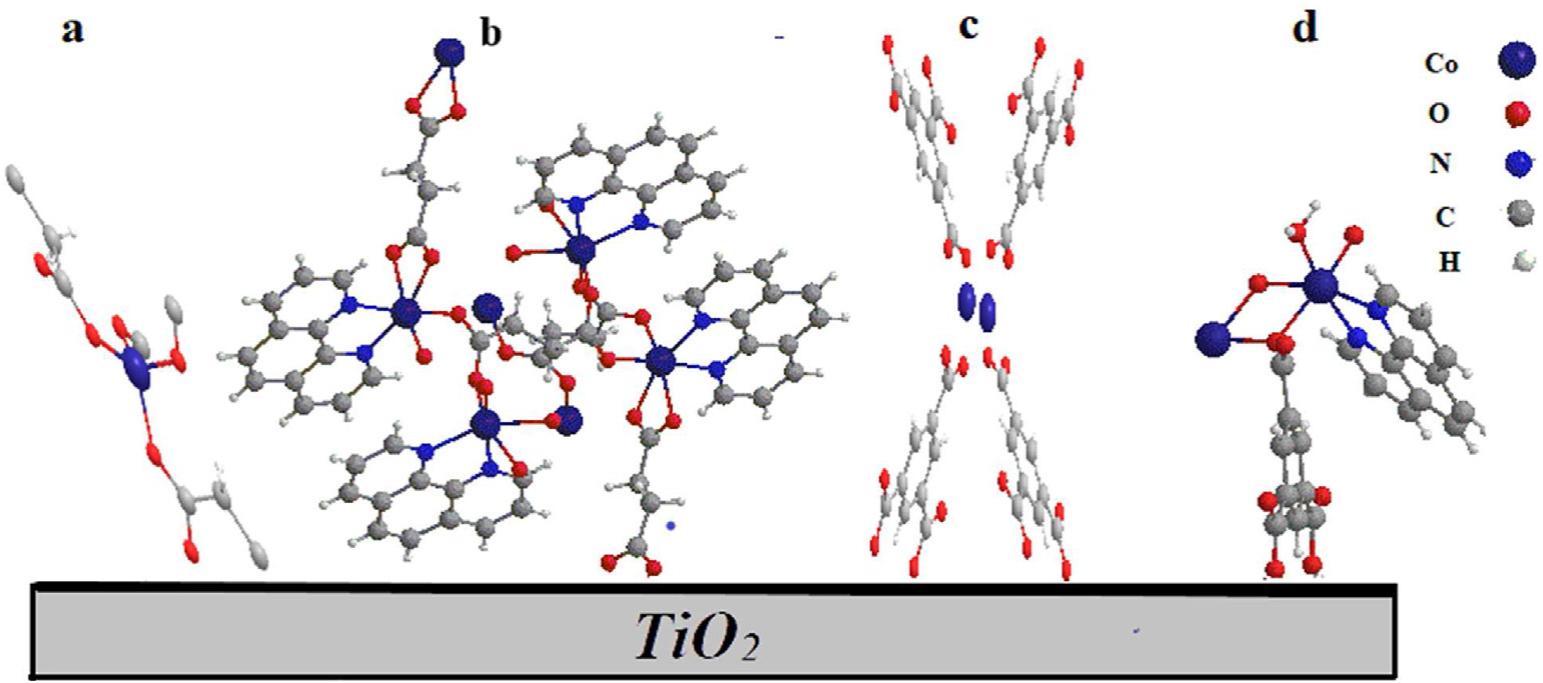
The coordination views and X-ray crystallographic structures of (a) complex 1, (b) complex 2, (c) complex 3 and (d) complex 4.

The important facture to consider in dye design is the role of functional groups on the dye that allow efficient adsorption on the semiconductor surface, and promote electronic communication between the acceptor orbitals of the semiconductor (TiO_2_) and the donor orbitals of the dye. The role of the carboxylate groups is to anchor the dye to the titania surface Figure 6(a-d).

### 3.7 Characterization of DSSCs

The photovoltaic performance of the cells was measured via radiation under simulated AM 1.5 G illumination of 1000 W cm^−2^. The performance parameters of devices, including fill factor (FF), short circuit current density (Jsc), open-circuit voltage (Voc), conversion efficiencies (η) were measured using the J-V curves according to Eqs. (2) and (3):

(2) $$FF=\frac{Im\times Vm}{JSC\times VOC}$$

(3) $$\eta =\frac{Pmax}{Pin}=\frac{Im\times Vm}{Pin}=\frac{ISC\times VOC\times FF}{Pin}$$

#### 3.7. 1 Photovoltaic analysis

**Figure 7:**
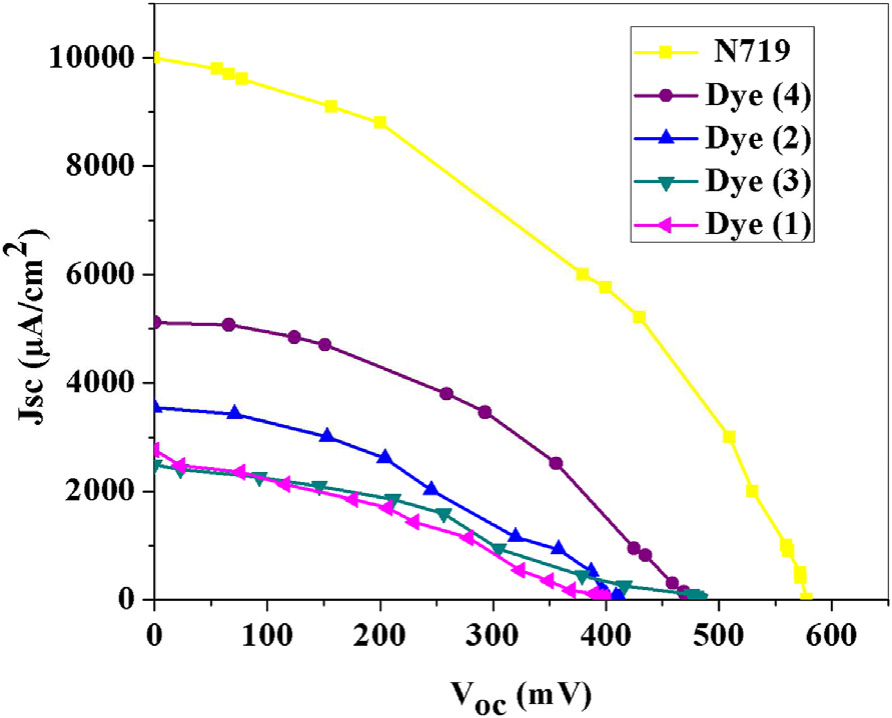
J–V curves of dyes (1–4) and N719 sensitized solar cells.

To investigate the photovoltaic performance of DSSCs (1–4), prepared cells were assembled with an architecture of FTO/TiO_2_/Dyes (1–4)/electrolyte/Pt/FTO. J-V characteristics of DSSCs (1–4) are shown in Figure 7. The experimental results of the fabricated devices, Voc, Jsc, FF, and η, are provided in Table 2. They have also been compared with the solar cell based on dye N719. The DSSC 1 produces a PCE of 0.35%, with a Jsc of 2760 μA cm^−2^, V_OC_ of 400 V, and FF of 0.316%. The addition of 1,10-phenantroline ligand as donating group improves the cell 2 performance, leading to J_SC_ of 3540 μA cm^−2^, V_OC_ of 413 V, FF of 0.35%, and PCE of 0.511%. A J_SC_ of 2484 μA cm^−2^, a V_OC_ of 483 V, FF of 0.33%, and a higher PCE of 0.395% are achieved by adding a carboxylate group by changing the succinic ligand to BTC in a dye structure of 3. The easy binding to the surface of TiO_2_ of the –OH groups in the BTC structure made the dye loading amounts of BTC-based dyes higher than those of suc-based dyes. The highest PCE of 1.006% is obtained from DSSC 4, due to the existence of both BTC and Phen ligands in dye structure. In complexes with phen ligands (2, and 4), electron recombination is less because the HOMO orbital level of dye is closer to the redox couple potential value (−4.85 eV).

**Table 2:** Photovoltaic parameters of solar cells with a photo-anode of TiO_2_/dyes: (1) [Co(suc)]_n_, (2) [Co_2_(suc)_2_(phen)_2_], (3) [Co_3_(BTC)_2_(H_2_O)_n_]_n_, and (4) [Co(HBTC) (phen) (H_2_O)_2_] and N719.

Dye	J_SC_ (μA/cm^2^)	V_OC_ (mV)	FF	(%) ƞ
1	2760	400	0.316	0.35
2	3540	413	0.350	0.511
3	2484	483	0.330	0.395
4	5116	480	0.410	1.006
N719	10000	578	0.4	2.30

#### 3.7.2 Incident photon-to-current efficiency (IPCE) measurements

**Figure 8:**
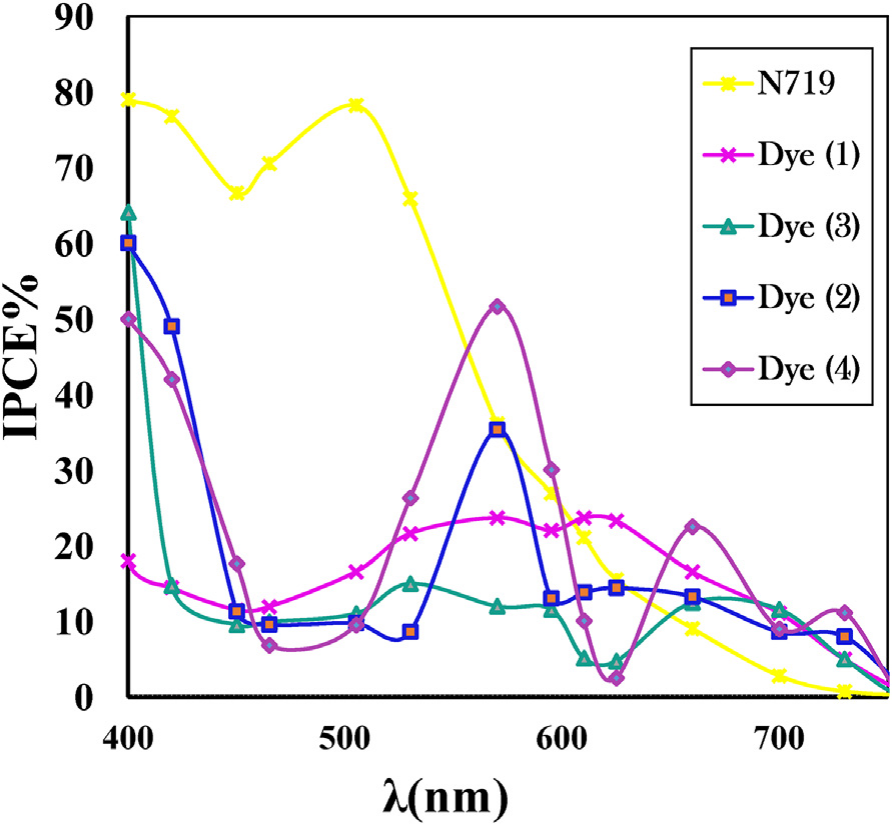
IPCE values of dyes (1–4) and N719 sensitized solar cells.

The spectra of the monochromatic incident photon to current efficiencies (IPCEs) for the dyes (1–4) and N719 as sensitizers in the 400–700 nm wavelength range, are shown in Figure 8. The IPCE value of the solar cell based on dye 4 is significantly higher than the other three dyes, mainly due to the relatively large Jsc compared with the other three dyes. The trend of the IPCE curve is consistent with the Jsc order of DSSC, and it is the same as that of the UV absorption spectrum. The IPCE spectrum of the solar cell based on dye 4 in the 570–700 nm region is intensified and extended to the near-IR compared to N719 dye.

### 3.8 Electrochemical impedance spectroscopy (EIS) measurement

**Figure 9:**
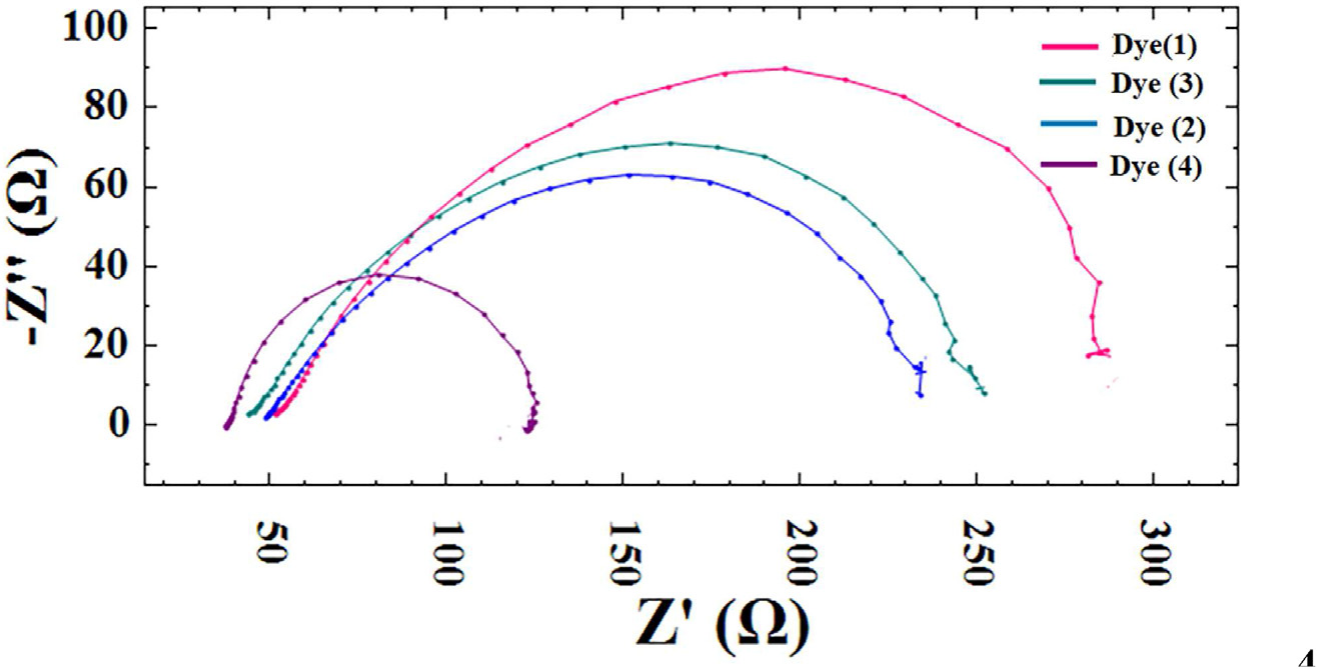
Nyquist curves of DSSCs based on dyes (1–4).

EIS is used to investigate the effect of the production of [Co(suc)]_n_(1) [Co_2_(suc)_2_(phen)_2_](2) [Co_3_(BTC)_2_(H_2_O)_n_]_n_(3) [Co(HBTC) (phen) (H_2_O)_2_](4) complexes on the devices interfacial charge transport behavior. In Figure 9, the Nyquist plots of devices based on different photoactive layers were measured and compared. The charge-transfer resistance)R_CT_ (is the resistance of the interface between the photoanode, dye, and electrolyte. The series resistance (R_S_) corresponds to the starting point of the curve, and R_S_ is the resistance of the FTO coated glass sheet. In Table 3, the R_S_ value of the dye (4) has the lowest value among the R_S_ of other dyes, which corresponded to the highest value of FF (Table 2). As can be seen in Figure 9 and Table 3, the R_CT_ resistance values were found as different with changing the ligand for dyes. The dye (4) system has the lowest R_CT_ resistance, and this dye system exhibited the highest conversion efficiency values at the same time (Table 2), which could be explained by more electrons being generated.

**Table 3:** EIS results for the DSSCs based on dyes (1–4).

Dye	R_s_ (Ω)	R_ct_ (Ω)
1	51	231.93
2	48.81	184.19
3	43.80	207
4	37.50	87.83

## 4 Conclusion

In this work, we synthesized four complexes (1–4) of cobalt (ІІ) as stable photosensitizers, and they were deposited on TiO_2_ surface by the facile immersing method to assemble DSSCs. The DSSCs performance is investigated in the presence and absence of an electron-donating ligand (phen) (2, 4), and (1, 3), respectively. The results exhibit relatively higher conversion efficiencies of dyes (2, 4) of 0.511%, and 1.006%, compared with device efficiencies of 0.35% and 0.395% for dyes (1, 3). The reason for this high efficiency is the stability of complexes with phen and their higher absorption in the UV-Vis light region. More electrons are transferred from the HOMO to the LUMO when the intensity of the absorbed sunlight band increases, and it improves the cell efficiency. The proximity of the HOMO to the potential energy level of redox electrolyte also affects the efficiency of the cell.

In addition, the effect of coordinated group numbers or binding sites, suc (two binding sites- 1, 2) and BTC (three binding sites-3, 4), on the performance of the cells was investigated. When the number of carboxylate group increases, the PCE achieves an improvement of 0.395% and 1.006% for dyes (3, 4) in comparison with (1, 2), 0.35% and 0.511%. As the results demonstrate, complex 4 has a higher efficiency because the two uncoordinated carboxylate groups have rather strong bonding with TiO_2_ nanoparticles. In conclusion, this work not only confirms the effectiveness of phen and BTC ligands anchoring Co(II)-dyes on the titania surface, but more importantly it demonstrates that it is possible to fabricate environmentally friendly cobalt-based DSSCs. In addition, this study provides new perspective into the use of transition metal complex dyes as viable alternatives for solar cells based on noble metal complex dye.

## Data availability

All data generated or analyzed during this study are included in this published article.

## Declarations

### Author contribution statement

Faezeh Arjmand: Performed the experiments; Analyzed and interpreted the data.

Zohreh Rashidi Ranjbar: Conceived and designed the experiments; Analyzed and interpreted the data; Contributed reagents, materials, analysis tools or data; Wrote the paper.

Hasan Fatemi Emam Gheiss: Analyzed and interpreted the data.

### Funding statement

This work was supported by the Shahid Bahonar University of Kerman.

### Data availability statement

Data included in article/supplementary material/referenced in article.

### Declaration of interests statement

The authors declare no conflict of interest.

### Additional information

No additional information is available for this paper.
