# Supplementary material for: Effect of dye complex structure on performance in DSSCs; An experimental and theoretical study
Source: Heliyon. 2022 Nov 17;8(11):e11692. doi: 10.1016/j.heliyon.2022.e11692 (PMC9679391; doi:10.1016/j.heliyon.2022.e11692)
Supplement: Supporting Information [file mmc1.docx]

**Supplementary Information**

**Effect of dye complex structure on performance in DSSCs; An experimental and theoretical study**

###

**Faezeh Arjmand ^a^, Zohreh Rashidi Ranjbar^a^*** **Hassan Fatemi E. G.^b^**

^a^ Department of Chemistry, Faculty of Sciences, Shahid Bahonar University of Kerman, Kerman, Iran;

^b^ Faculty of Physics, Shahid Bahonar University of Kerman, Kerman, Iran

*Email: zoh.rashidi@uk.ac.ir, zoh.rashidi@gmail.com; ORCID No: 0000-0002-8152-9736

Fig. S1 Tauc plots (a-d) showing indirect band gap values of dyes (1-4).
